# KIFC1 inhibition: Exploring the potential of propolis-derived small molecules for targeting cancer progression through *in silico* analysis

**DOI:** 10.1371/journal.pone.0324678

**Published:** 2025-06-05

**Authors:** Muhammad Bilal Azmi, Simran Kumari, Sakina Aquil, Urooj Nizami, Arisha Sohail, Syed Danish Haseen Ahmed, Shamim Akhtar Qureshi

**Affiliations:** 1 Computational Biochemistry Research Laboratory, Department of Biochemistry, Dow Medical College, Dow University of Health Sciences, Karachi, Pakistan; 2 Dow Medical College, Dow University of Health Sciences, Karachi, Pakistan; 3 Department of Biochemistry, University of Karachi, Karachi, Pakistan; Universidad San Francisco de Quito - Campus Cumbaya: Universidad San Francisco de Quito, ECUADOR

## Abstract

Propolis, a resinous compound produced by bees, possesses diverse medicinal properties and has gained significant attention for its potential in cancer therapy. This study investigated the therapeutic significance of propolis-derived compounds targeting the kinesin-like protein KIFC1, a motor protein overexpressed in various cancers, using a multistep computational methodology. Therefore, it is essential to utilize different *in silico* methods to predict their therapeutic potential. A 3D library of propolis-derived compounds sourced from previously published literature was compiled and screened for physicochemical properties, drug-likeness, and pharmacokinetic predictions using the SwissADME and BOILED-Egg permeation predictive model. Pharmacokinetic computations were used to filter out compounds that lacked drug-likeness attributes. KIFC1 3D homology model was selected from the AlphaFold database, its stereochemical properties were assessed and validated. Virtual screening was performed to identify the high-binding affinity-based top-ranked compounds. Furthermore, the active residues present in the druggable cavities were identified using the Cavity Blind (CB) docking tool to investigate grid-box-based residue-specific molecular docking and simulation analysis. We found five common propolis-derived compounds following the druglikeness rule, and having HBA (high binding affinity) for the KIFC1 protein, were subjected to CB docking to identify druggable binding pockets (recognition of consensus residues) on KIFC1 as well as residue-specific molecular docking and simulation. Grid-box-based docking experiments for exploring the molecular interactions of the five compounds above validated the inhibitory effects of kaempferide (∆G = −7.35 kcal/mol and *K*_*i*_ = 4.12 μM), luteolin (∆G = −6.74 kcal/mol and *K*_*i*_ = 11.48 μM), Izalpinin (∆G = −6.33 kcal/mol and *K*_*i*_ = 22.9 μM), 4’,5,7-Trihydroxy-3,6-dimethoxyflavone (∆G = −6.14 kcal/mol and *K*_*i*_ = 31.71 μM), and 6-methoxykaempferol (∆G = −6.55 kcal/mol and *K*_*i*_ = 15.81 μM). Molecular dynamics simulation analysis at 100 nanoseconds examined the binding modes of five screened compounds and predicted molecular interactions with KIFC1 protein residues. Two propolis-derived compounds, 4’,5,7-trihydroxy-3,6-dimethoxyflavone and 6-methoxy kaempferol, showed significant interactions with KIFC1 residues and exhibited stable binding pattern. MD simulations analysis showed minor variation in root-mean-square deviation and fluctuation, confirming their equilibrium with KIFC1 protein. The study enhances understanding of propolis compounds’ inhibitory effects on KIFC1 protein, providing insights for potential treatment approaches and requiring further experimental (*in vivo* and *in vitro*) as well as clinical validation.

## Introduction

The chemical substances obtained from bees, such as honey, royal jelly, and propolis, have innate characteristics that have been involved in the management of countless diseases for years[1]. Propolis is a resinous substance formed by honey bees and has been used in traditional medicine for its antibacterial, antiviral, antifungal, and antiparasitic characteristics. Notably, its antioxidant and antiproliferative properties offer its role in anticancer therapy[2–6]. Honey bees produce propolis by collecting resins, saps, and other materials from plants and combining them with beeswax and other proteins/enzymes[7]. The chemical constituents of propolis differ by plant species and are influenced by regional and environmental features[8–10]. More than 300 compounds have been identified, including aromatic acids, aliphatic compounds, flavonoids, terpenes, sugars, esters, macro as well as micronutrients, and vitamins[3,8,9]. Polyphenols as well as terpenoids are the most active compounds in propolis [10] and have been shown to modulate the cell cycle, arrest cancer cell proliferation, and inhibit angiogenesis[11]. Certain types of propolis, such as Chinese and Brazilian red propolis, have exhibited efficacy against various cancer cell lines by inhibiting the pathways linked to inflammation and cell proliferation[11–14]. Globally, the burden of cancer incidence and mortality is rapidly growing. According to a World Health Organization (WHO) report in 2019, cancer was the most important or second leading cause of death in 112 of 183 nations before the age of 70. In 2020, 19.3 million new cases were diagnosed worldwide and 10 million cancer-related deaths were reported, with 50% of the new cases and 58.3% of cancer-related deaths occurring in Asia[15]. By 2040, 28 million new cases of cancer are expected per annum if the current trends continue.

Recent studies have reported prominent KIFC1 expression in various cancers, including breast, prostate, ovarian, and non-small cell lung cancers, making it a promising target for cancer therapy owing to its redundancy in normal somatic cells[16,17]. Data indicate that KIFC1 expression correlates with higher Gleason scores, increased metastatic potential, and other markers of cancer progression, emphasizing its potential as a therapeutic target[18–21]. KIFC1 belongs to the kinesin-14 and is a C-terminal kinesin characterized by minus-end motility along microtubules[22,23]. It plays a distinctive role in mitosis, meiosis, vesicular and organelle transport, spermiogenesis, and oocyte development[24,25]. Structurally, KIFC1 consists of three domains: a motor domain, stalk domain, and a microtubule-binding domain, similar to other motor proteins[26]. The motor contains the ATP hydrolysis site and microtubule interaction region, generating the energy required for spindle elongation, stability, and organization[27,28]. A fundamental function of KIFC1 is its role in mitotic spindle assembly and chromosomal stability, which are critical for cell division and cell survival. During mitosis, proper spindle formation ensures accurate chromosomal segregation, thereby preventing aneuploidy and genomic instability. In cancer cells with supernumerary centrosomes, KIFC1 prevents multipolar mitotic spindle formation, which otherwise leads to mitotic catastrophe and cell death [25,29]. This is achieved by crosslinking and sliding microtubules, facilitating centrosome clustering, and stabilizing bipolar spindle formation [25,30]. Additionally, KIFC1 interacts with key mitotic regulators, such as cyclins (Cyclin B1, Cyclin D, and Cyclin A), spindle assembly checkpoint proteins (MAD1-MAD2), and Aurora B kinase, ensuring successful mitotic progression and promoting uncontrolled proliferation in cancer cells [25,30–32]. The ability of KIFC1 to rescue chromosomal instability in highly proliferative tumor cells further supports its relevance as a cancer therapeutic target [23,25,30,33]. Several small-molecule inhibitors that interact with KIFC1 and disrupt its function have been identified, effectively reversing the centrosome clustering phenotype in cancer cells [34,35]. However, obtaining structural data to precisely map the binding sites of KIFC1 inhibitors remains a challenge. Further research is required to refine these inhibitors and improve their specificity and drug-like properties to enhance their therapeutic potential.

Virtual screening and molecular dynamics simulations have identified a few promising small-molecule inhibitors with strong binding interactions with KIFC1[36]. The KIFC1 inhibitors structure and binding data across various small-molecule inhibitors remain limited. Therefore, this study used a comprehensive docking analysis of propolis-derived small molecules or compounds to computationally target the inhibitory activity of KIFC1 protein expression. This computational analysis revealed the binding affinities of propolis-derived compounds at three possible binding sites in the motor domain. Docking analysis was also helpful for identifying potential KIFC1 inhibitors and contributing to the drug design process for KIFC1 inhibitors. Therefore, this study may be fundamental for the development of novel anticancer compounds from propolis-derived small molecules with increased potency, improved pharmacological profiles, and low toxicity.

## Methodology

**Fig 1:**
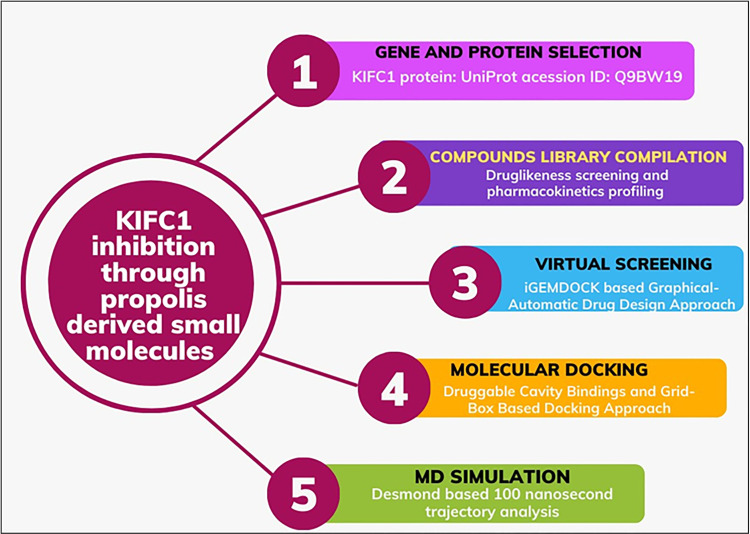
Stepwise schematic workflow of the present study.

**Fig 1** shows the step-wise scheme of the present work methodology.

### Retrieval of the gene and protein sequence of KIFC1

The gene for the kinesin-like protein (*KIFC1*) encodes a minus end-directed motor protein was retrieved from the UniProt database (https://www.uniprot.org/)[37,38]. The authenticity of the gene sequence was confirmed based on annotation status, information gathered from literature and curator-reviewed computational analysis [38]. Gene identifiers and NCBI accession numbers were cross-referenced to validate the obtained gene information (https://www.ncbi.nlm.nih.gov/gene/3833) [39].

The FASTA format of selected gene was obtained from the NCBI database and the Basic Local Alignment Search Tool (BLAST) (https://blast.ncbi.nlm.nih.gov/Blast.cgi) compared the human protein sequence (query sequence) against target sequences in the database by selecting research collaboratory for structural bioinformatics (RCSB) protein data bank (PDB) (https://www.rcsb.org/search/advanced/sequence) as a set parameter to conduct a protein BLAST search of the relevant collection[40,41].

### 3D homology model of the KIFC1 protein and structural validation

The AlphaFold Protein Structure Database, which is accessible via UniProt database (https://alphafold.ebi.ac.uk), was employed to select the optimal 3D protein homology model for comprehensive structural coverage of amino acid residues in the canonical protein-coding sequence (canonical) of the KIFC1 gene [42,43]. The purpose of employing this strategy was due to the unavailability of complete 3D protein model in PDB. If the sequence query coverage was not complete, a 3D model of target protein with complete coverage (amino acid length 673) from the canonical sequence was chosen as the preferable option. AlphaFold is recognized as the leading protein structure prediction method in the Critical Assessment of Techniques for Protein Structure Prediction (CASP14) by a significant margin, generating highly accurate predictions. This database is an artificial intelligence (AI)-based system established by Google DeepMind and EMBL’s European Bioinformatics Institute (EMBL-EBI), which uses the amino acid sequence of protein to predict the 3D structure. This tool regularly achieves accuracy competitive with experiments [44].

After selecting 3D protein model, UCSF Chimera https://www.cgl.ucsf.edu/chimera/ was used to investigate the molecular characteristics and visualize the resulting protein structure [45] while the energy of KIFC1 PDB structure was minimized through Swiss PDB Viewer tool (https://spdbv.unil.ch/) [46]. The model built was validated through PROCHECK https://www.ebi.ac.uk/thornton-srv/software/PROCHECK/ using an ERRAT quality factor, Ramachandran plot, Prosa-web (https://prosa.services.came.sbg.ac.at/prosa.php), and the residual properties of the constructed model [47]. The dihedral angles φ against ψ of the possible conformations of amino acids in the protein structure were also studied in the Ramachandran plot [48,49]. The structure validation server – SAVES (https://saves.mbi.ucla.edu/) was used to determine the probable structural errors and z scores of the selected 3D protein model.

### Propolis derivatives: three-dimensional compound library compilation

The propolis-derived small molecules were sourced from previously published literature and their three-dimensional configurations were acquired from the PubChem database (https://pubchem.ncbi.nlm.nih.gov) [50] in the Structure Data Format (SDF) format. The SDF-2D format of all compounds were converted into PDB-3D format using Open Babel software https://openbabel.org/index.html and visualized using the BIOVIA Discovery Studio Visualizer (https://www.3ds.com/products/biovia/discovery-studio)[51].

### Assessment of propolis derivatives: physicochemical properties, pharmacokinetics and drug likeness

The propolis derivatives were analyzed to predict their pharmaceutically important descriptors (ADME) and physiochemical attributes. The physicochemical properties of compounds include molecular weight (MW), number of heavy atoms (HA), number of aromatic heavy atoms (ARO HA), fraction of carbons in the sp³ hybridization (FCsp³), rotatable bond (RB), hydrogen bond acceptor (HBA), hydrogen bond donor (HBD), molar refractivity (MR), topological polar surface area (TPSA), pharmacokinetics properties including GI absorption, BBB permeation, P-gp substrate status, cytochrome-P enzyme inhibition, and skin permeation (log Kp), for predicting drug absorption and distribution within the body as well as drug likeness (based on Lipinski’s rule of five), were predicted using SwissADME (http://www.swissadme.ch/) [52]. The physiochemical properties such as lipophilicity (logP), skin permeation and bioavailability score for drug-likeness were presented numerically, while the remaining pharmacokinetic and drug-likeness parameters were shown categorically. The solubility parameters of propolis derivatives such as, logP and logS values were predicted using pkCSM (https://biosig.lab.uq.edu.au/pkcsm/) [53]. A boiled egg prediction model was constructed using the topological surface area (TPSA) and the lipophilicity (logP) of propolis compounds to analyse gastrointestinal tract absorption and blood-brain barrier penetration [52].

### Integrated virtual screening of propolis-derived small compounds: exploring druggable binding pockets with a residue-specific grid-box-based molecular docking approach

The KIFC1 protein structure used in this study was obtained from the AlphaFold protein structure database [42–44]. However, detailed characterization of the active site residues and binding pockets is not readily available because of the inherent limitations of the predicted 3D structural model and the absence of a fully resolved structure in the PDB repository. Given this constraint, we employed a blind docking approach, a widely accepted computational strategy in drug discovery, particularly when the exact binding site is unknown or not well-defined [54–56]. This method allows for unbiased identification of potential binding sites and facilitates the screening of promising inhibitors for further experimental validation [55]. Moreover, our primary objective in utilizing this strategy was to enhance the efficiency of propolis-derived small molecule screening by rapidly identifying potential candidates (by narrowing the pool of compounds) for subsequent validation studies.

Virtual screening of propolis-derived small molecules were conducted through iGEMDOCK (http://gemdock.life.nctu.edu.tw/dock/igemdock.php) to narrow the number of candidate compounds for further computational validation [54]. Blind docking approach was used in this virtual screening because the active site information of a target protein is not available. This method of computational analysis aids in the identification of potential binding regions on protein and candidate compounds. The identified top-ranked compounds were subjected to hit identification testing using the iGEMDOCK graphic automatic drug design system to determine the ligand-protein associations based on the fitness values. iGEMDOCK is a graphical-integration platform for virtual screening that employs k-means and hierarchical clustering techniques for analyzing compound characteristics and protein-ligand interactions. It provides novel post analysis tools, such as atomic composition (AC), which assesses compound similarity by comparing it to the amino acid present in a protein sequence [54].

Among the forty screened compounds, the five with the highest binding energies were selected for further optimizations using cavity detection-guided blind docking https://cadd.labshare.cn/cb-dock2/ approach [55,56]. The cavity binding docking (CB dock) method generated consensus interaction cavities within the 3D model of KIFC1 protein after docking it with the selected top-ranked screened drug-like compounds of propolis. An automated protein-ligand docking approach was used to conduct CB-guided blind docking to examine binding cavities and sites within the homology model. CB Dock utilizes “CurPocket” approach to compare and rank cavities. This method applies protein–ligand binding site prediction techniques, using the COACH benchmark set as reference [57]. This utilizes a novel method of cavity detection based on their curvature. Moreover, it determines the center and dimensions of the docking box of a potential cavity as a crucial parameter of the process. This approach was meticulously optimized, achieving a success rate of 70% in the top-ranking poses, with a root mean square deviation (RMSD) within 2 Å from the X-ray pose for a test set of structures, not specifically for the presently investigated protein [56].

We further scrutinized the cavities with the highest grades reflecting the consensus residues generated after interaction of each propolis compound with the KIFC1 protein model for molecular visualization and interpretation of the therapeutic intervention using a 2D ligand plot. The consensus-interacting residues identified through CB Dock were documented and utilized for grid-box-based AutoDock Vina (version 4.2) https://autodock.scripps.edu/download-autodock4/ analysis of protein‒propolis interactions [58,59]. The polar hydrogen was incorporated, and partial charges were assigned to the standard residue using the Gasteiger partial charge method, which assumes that all hydrogen atoms are explicitly represented. The most favorable binding interactions were estimated based on the lowest predicted binding free energy obtained from the best molecular docking simulation pose [59]. The inhibition constant (*K*_*i*_) was derived from the binding energy (ΔG) using the formula *K*_*i*_ = exp(ΔG/RT), where R is the universal gas constant (1.985 × 10 ^−3^ kcal mol ^−1^ K ^−1^) and T is the temperature (298.15 K) [60].

### Molecular dynamics simulation

The MD simulations were performed using the Desmond simulation package to further optimize the top five propolis compounds as a promising lead compound [61–63] in the physiological environment. The system was constructed using the TIP3P (Intermolecular Interaction Potential 3 Points Transferable) tool and the ligands and the protein were prepared using an orthorhombic box with the OPLS_2005 force field as the solvent model [64,65]. The simulation was performed at 300 K (temperature) and 1 atm (pressure), and the model was neutralized by adding 0.15 M sodium chloride. The protein-ligand complex trajectories were saved every 100 picoseconds (ps) and visualized using the simulation interaction diagram tool in the Desmond package (Schrödinger Release 2021−2: Desmond Molecular Dynamics System DESR. New York). The stability dynamics of the protein‒ligand complex were determined by measuring the root mean square deviation (RMSD) and root mean square fluctuation (RMSF) over 100 ns [66]. In molecular modeling and structural analysis, RMSD is a measure of the average atom displacement between two superimposed molecular structures, to evaluate conformational differences [63,66]. Principal Component Analysis (PCA) was utilized to examine large-scale conformational dynamics in molecular dynamics (MD) simulations by reducing the dimensionality of atomic displacement data and extracting dominant motion patterns [67,68]. This statistical approach effectively differentiates functionally significant biomolecular motions from random fluctuations, providing a comprehensive visualization of the conformational landscape. By identifying key structural transitions, PCA facilitates comparative assessments between molecular states, offering insights into protein flexibility, allosteric modulation, and ligand-induced conformational changes. The analysis was conducted using the Bio3D package in R [69], where a dedicated script written in R was used to compute the principal components and evaluate the essential motions of the system [67,68].

### MM-GBSA calculations

The Molecular Mechanics Generalized Born Surface Area (MM-GBSA) Gibbs free energy change (ΔGbind) calculations, along with molecular docking and MD simulations, were collectively employed to identify the most stable KIFC1 inhibitors (propolis-derived small molecules). This approach addressed the limitations of overestimated binding affinities in docking studies and the lack of quantitative scoring in MD simulations [70]. The MM-GBSA method was utilized to compute the binding free energy (ΔGbind). The ‘MM-GBSA’ module in Maestro 12.3 was used for these calculation, employing the OPLS-2005 force field and the VSGB 2.0 energy model as the solvent model. All other settings were applied using default parameters. The resulting binding free energies were measured in kcal/mol. The MM-GBSA binding free energies were determined using the following formula:


$$\Delta {𝐆}_{bind}={𝐆}_{complex}-{𝐆}_{receptor}-{𝐆}_{ligand}$$

Including all interactions between the ligand and protein, the free energy of the protein-ligand complex is represented by the term G_complex_. It considers the contributions of bonded (covalent) and nonbonded interactions, including solvation effects, hydrogen bonding, van der Waals forces, and electrostatic interactions. G_receptor_ is the free energy of an unbound protein receptor that has been isolated. This signifies the energy linked to the structure of the protein and the interactions that occur within the protein, instead of with the ligand. The unbound free energy of the isolated ligand is known as the G_ligand_, which refers to the ligand structure and its internal interactions, excluding those with the protein.

## Results

### *KIFC1* gene, protein selection and its 3D structure-template search

The details of the *KIFC1* gene and protein were obtained from NCBI and UniProt using gene ID 3833 and accession number Q9BW19. The reference sequence NC_000006.12 was utilized to select an appropriate protein for molecular docking. The KIFC1 gene has four transcripts with a total of 2694 base pairs, situated at 6p21.32 with 13 exons. Notably, the gene results were similar to HSET and KNSL2 genes. The molecular weight of KIFC1 protein is 73,748 Da and is made of 673 amino acids with Leucine being the most abundant amino acid. The protein has net positive charge and half-life in mammalian reticulocytes is 30 hours.

The BLAST results of the selected KIFC1 protein sequence identified several sequences that matched the 3D structures in the PDB database (S1 Table**).** The crystal structure of PDB ID 5WDH_A was the best match with maximum score of 731, a sequence length of 376 residues, 53% query coverage, and 99.72% identity. However, the preference was given to selecting a PDB model that provided full coverage and complete residue similarity with the chosen protein sequence. Therefore, the AlphaFold database was preferred to obtain a protein sequence specific, complete 3D model. Currently, this database is preferred to generate a 3D structure with complete identical sequence information for the protein target. Subsequently, the structural identifier ‘AF-Q9BW19-F1’ of the desired protein encoded by the *KIFC1* gene was used to obtain its 3D model from the AlphaFold database. Before further use, the structure was meticulously cleaned to remove any heteroatoms, ions, or other bound molecules. This preparation was essential to enhance the interactions and generalizability of the ensuing outcomes.

### KIFC1 3D structure validation and refinement

The 3D structure of KIFC1 protein retrieved from AlphaFold database was downloaded in the PDB format and assessed for its stereochemical quality and structural accuracy. The PROCHECK analysis revealed in the Ramachandran plot that 91.4% of the residues were in the core region, 6.7% were in the allowed zone (yellow color), and 1.2% were in the generously allowed zone (red color). The ERRAT quality factor was 92.5%. The bond/length angle was 10.2, with the highest deviation being 18. There was no reported bad contact score according to the 3D structural residue attributes (S1 Fig.). The 3D structural G-factor analysis by SAVES showed a total value of −0.01, a dihedral value of −0.12, and a covalent value of 0.15. In addition, the planar group analysis revealed that 6.3% of the residues were highlighted and 93.7% of the residues were within limits. The energy of the KIFC1 protein structure was further refined by minimizing it to −37642.84 kJ/mol. This process resulted in 937.473 bonds, 3229.849 angles, and 4133.502 torsion events. Additionally, the electrostatic energy was −28286.47. The energy minimization of the homology model increased the overall quality factor to 94.80%.

### *In silico* prediction of potential pharmacokinetic and drug-like properties of propolis compounds

**Fig 2:**
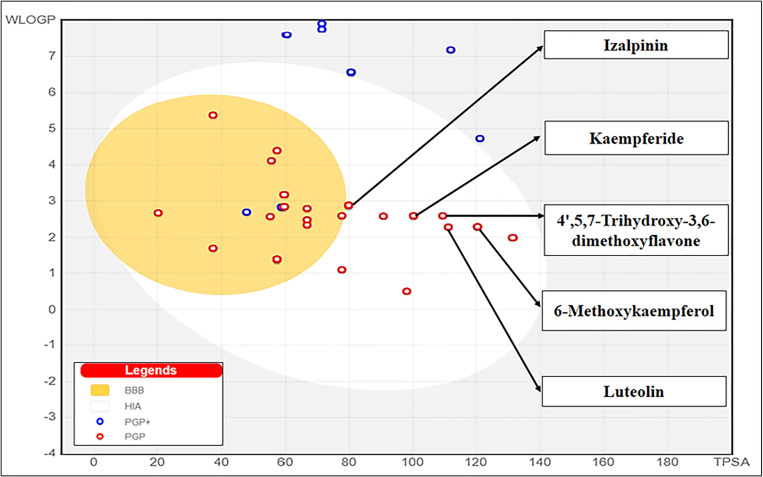
BOILED-Egg plot representing the intestinal absorption and brain penetration potential of propolis-derived compounds. The white region indicates compounds predicted to be well absorbed in the intestine, while the yellow region signifies those likely to cross the blood-brain barrier. Compounds in the gray region are predicted to have poor intestinal absorption and limited brain penetration. Red and blue dots represent P-gp-negative and P-gp-positive compounds, respectively.

**Fig 3:**
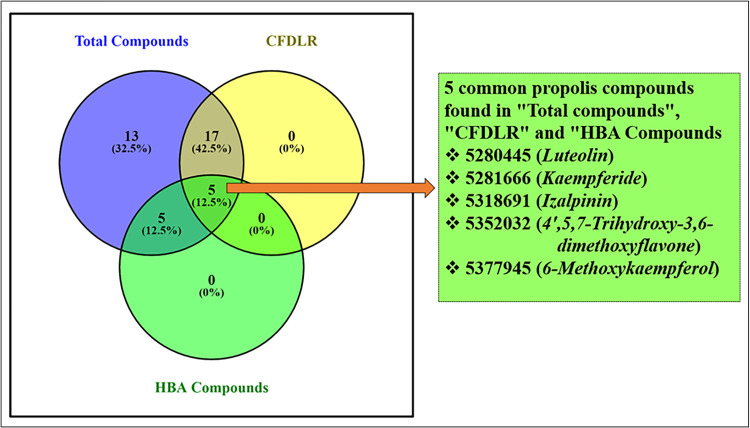
Venn diagram illustrating the overlap between propolis-derived compounds that comply with the criteria for drug-likeness rules (CFDLR) and those exhibiting strong binding energies (HBA) towards the KIFC1 protein.

The canonical SMILES and structures of propolis compounds were deposited in PDB format in the library for physiochemical screening (S2 Table). The molecular weight, number of heavy and aromatic heavy atoms, fraction of carbons in the sp³ hybridization, rotatable bonds, hydrogen bond acceptor and bond donor, molar refractivity and topological polar surface area of compounds were screened and compounds with PUBCHEM IDs 44257510, 5472440, 637125, 336327, 92503, 72307, and 6549 were identified within the desirable range out of forty compounds (**Table 1**). The lipophilicity parameters such as XLogP3, WLogP and MLogP of thirty propolis compounds were within the desirable limits showing balance of hydrophilicity and lipophilicity suggesting that the compounds likely have favorable ADMET properties (**Table 2**). The solubility (Log S) parameters such as Estimated Solubility (ESOL), solubility methods developed by Ali and SILICOS-IT [Log S (Ali) and Log S (SILICOS-IT)] were used to quantify water solubility of propolis compounds and classified them into insoluble, poorly, moderately, soluble, very or highly water soluble. Nine propolis compounds were predicted to likely have desirable water solubility by all three solubility parameters suggesting good absorption, bioavailability, and overall efficacy as drug candidates (**Table 3).** The skin permeability, Kp, values predicted for all the propolis compounds were in the range of −1.9 to −6.9 cm/s indicating low skin permeability (**Table 4**). This study found that eleven out of forty propolis compounds can act as drug transporters for P-glycoprotein substrate (**Table 4**) which could potentially result in drug-drug interactions, accumulation of drugs or their by-products and toxic or other undesired effects due to reduced clearance. The inhibition of CYP enzymes is a common factor in drug interactions. However, CYP3A4 inhibitors have the potential to enhance the effectiveness of specific chemotherapy drugs by increasing their plasma concentrations and bioavailability. As indicated in **Table 4**, all propolis compounds exhibited CYP3A4 inhibitory effects except for 11 compounds. Thirteen out of the forty propolis compounds violated Lipinski’s rule of five for molecular weight. Overall, twenty-two compounds adhered to all five drug-likeness criteria, with a bioavailability score exceeding 0.55, signifying their drug-like molecular nature (**Table 5**). In **Fig 2**, the prediction of intestinal absorption and blood-brain barrier penetration is given in the form of BOILED egg model. The yolk (yellow part) represents the compounds most likely to penetrate the BBB and the white region shows a greater propensity for HIA penetration while P-gp-positive and P-gp-negative molecules are denoted by blue and red dots. Of the forty compounds, eighteen compounds resided in the blood-brain barrier penetration area and thirty-one compounds were located in intestinal absorption area **Fig 2**. Eleven propolis compounds found to be P-gp positive (blue dots) were eliminated from the brain penetration region which excluded them from further optimization. The drug-likeness criteria (CFDLR) and higher binding affinity with the KIFC1 protein (HBA) led to select five promising compounds out of forty: 6-methoxy kaempferol, 4’,5,7-Trihydroxy-3,6-dimethoxyflavone, Izalpinin, Kaempferide, and luteolin (**Fig 3**). Only Izalpinin was predicted to penetrate the blood-brain barrier and be absorbed in the gastrointestinal tract due to its lower TPSA value. All five compounds showed similar bioavailability score (0.55) and were CYPIA2, CYP2D6 and CYP3A4 inhibitors in addition to 4’,5,7-Trihydroxy-3,6-dimethoxyflavone that acted as CYP2C9 inhibitor enzyme suggesting as suitable drug candidate for KIFC1 inhibition (**Table 4**).

**Table 1:** The compounds’ identifiers and physicochemical properties of propolis compounds.

S. No.	PubChem ID	MW (g/mol)	HA	ARO HA	FCsp3	RB	HBA	HBD	MR	TPSA (Å²)
1	44257510	272.3	20	12	0.25	2	4	2	75.62	58.92
2	21721815	324.37	24	12	0.25	4	4	1	92.77	55.76
3	11455669	552.7	40	6	0.61	7	7	3	153.79	121.13
4	11272353	518.68	38	6	0.55	7	5	1	150.95	80.67
5	11114020	518.68	38	6	0.55	7	5	1	150.95	80.67
6	10907594	534.68	39	6	0.48	7	6	3	156.5	111.9
7	10791588	502.68	37	6	0.55	6	4	0	149.79	60.44
8	9984117	518.68	38	6	0.55	7	5	1	150.95	80.67
9	5472440	300.39	22	6	0.32	6	3	2	92.57	57.53
10	5471610	502.68	37	6	0.48	7	4	1	152.45	71.44
11	5377945	316.26	23	16	0.06	2	7	4	82.5	120.36
12	5352032	330.29	24	16	0.12	3	7	3	86.97	109.36
13	5318691	284.26	21	16	0.06	2	5	2	78.46	79.9
14	5281954	268.26	20	16	0.06	2	4	1	76.44	59.67
15	5281787	284.31	21	12	0.12	6	4	2	80.77	66.76
16	5281666	300.26	22	16	0.06	2	6	3	80.48	100.13
17	5281628	300.26	22	16	0.06	2	6	3	80.48	100.13
18	5281616	270.24	20	16	0	1	5	3	73.99	90.9
19	5280681	316.26	23	16	0.06	2	7	4	82.5	120.36
20	5280445	286.24	21	16	0	1	6	4	76.01	111.13
21	5280442	284.26	21	16	0.06	2	5	2	78.46	79.9
22	5280373	284.26	21	16	0.06	2	5	2	78.46	79.9
23	5280343	302.24	22	16	0	1	7	5	78.03	131.36
24	3938139	244.24	18	12	0.07	3	4	2	66.85	66.76
25	3873459	246.26	18	10	0.21	3	4	1	69.75	59.67
26	689043	180.16	13	6	0	2	4	3	47.16	77.76
27	638278	256.25	19	12	0	3	4	3	72.32	77.76
28	637542	164.16	12	16	0	2	3	2	45.13	57.53
29	637541	164.16	12	6	0	2	3	2	45.13	57.53
30	637540	164.16	12	6	0	2	3	2	45.13	57.53
31	637125	302.45	22	0	0.65	4	2	1	93.86	37.3
32	637105	502.68	37	6	0.48	8	4	1	151.88	71.44
33	444539	148.16	11	6	0	2	2	1	43.11	37.3
34	336327	270.28	20	12	0.25	1	4	1	73.17	47.92
35	259846	426.72	31	0	0.93	1	1	1	135.14	20.23
36	238782	256.25	19	12	0.13	1	4	2	69.55	66.76
37	92503	272.3	20	12	0.25	2	4	2	75.62	58.92
38	72307	354.35	26	12	0.4	2	6	0	90	55.38
39	6549	154.25	11	0	0.6	4	1	1	50.44	20.23
40	370	170.12	12	6	0	1	5	4	39.47	97.99

***Abbreviations with reference range:*** MW: molecular weight (≤ 500 gm^-1^), HA: number of heavy atoms, ARO HA: number of aromatic heavy atoms, FCsp³: fraction of carbons in the sp³ hybridization (≥ 0.25), RB: rotatable bond (≤ 10), HBA: hydrogen bond acceptor (≤ 10), HBD: hydrogen bond donor (≤ 5), MR: molar refractivity (≤ 130), TPSA: topological polar surface area (≤ 150).

**Table 2:** The LogP values for lipophilicity of propolis compounds predicted using different computation methods.

S. No.	PubChem ID	Log Po/w (iLOGP)	Log Po/w (XLOGP3)	Log Po/w (WLOGP)	Log Po/w (MLOGP)	Log Po/w (SILICOS-IT)	Consensus Log Po/w
1	44257510	2.26	2.94	2.83	1.87	2.89	2.56
2	21721815	3.58	4.7	4.7	2.38	4.24	3.81
3	11455669	4.08	4.42	4.73	1.77	6.05	4.21
4	11272353	4.68	6.71	6.57	3.3	7.43	5.74
5	11114020	4.38	6.77	6.57	3.3	7.43	5.69
6	10907594	4	8.41	7.18	2.95	7.36	5.98
7	10791588	4.58	7.69	7.6	4.13	8.29	6.46
8	9984117	4.6	6.71	6.57	3.3	7.43	5.72
9	5472440	3.06	5.37	4.4	3.75	4.76	4.27
10	5471610	4.16	9.12	7.76	4.06	8.28	6.68
11	5377945	2.1	1.87	2.29	−0.31	2.06	1.6
12	5352032	2.56	2.82	2.59	−0.07	2.59	2.1
13	5318691	2.68	2.58	2.88	0.77	3.03	2.39
14	5281954	2.88	3.85	3.17	1.33	3.52	2.95
15	5281787	2.66	4.15	2.79	2.62	3.26	3.09
16	5281666	2.43	2.22	2.59	0.22	2.55	2
17	5281628	2.27	2.99	2.59	0.22	2.55	2.12
18	5281616	2.08	2.25	2.58	0.52	2.52	1.99
19	5280681	2	2.71	2.29	−0.31	2.06	1.75
20	5280445	1.86	2.53	2.28	−0.03	2.03	1.73
21	5280442	2.56	3.35	2.88	0.77	3.03	2.52
22	5280373	2.55	2.99	2.88	0.77	3.03	2.44
23	5280343	1.63	1.54	1.99	−0.56	1.54	1.23
24	3938139	2.12	3.43	2.34	1.41	2.43	2.34
25	3873459	2.79	3.02	2.84	1.79	3.04	2.7
26	689043	0.97	1.15	1.09	0.7	0.75	0.93
27	638278	2.02	3.18	2.59	1.58	2.48	2.37
28	637542	0.95	1.46	1.38	1.28	1.22	1.26
29	637541	1.14	1.79	1.38	1.28	1.22	1.36
30	637540	1.09	2.03	1.38	1.28	1.22	1.4
31	637125	3.14	5.71	5.37	4.45	4.94	4.72
32	637105	4.87	8.28	7.92	4.06	8.28	6.68
33	444539	1.55	2.13	1.68	1.9	1.7	1.79
34	336327	2.53	2.77	2.69	1.87	1.87	2.52
35	259846	4.68	9.87	8.02	6.92	6.82	7.26
36	238782	2.11	2.88	2.48	1.27	2.55	2.26
37	92503	2.26	2.94	2.83	1.87	2.89	2.56
38	72307	3.46	2.68	2.57	1.98	3.25	2.79
39	6549	2.7	2.97	2.67	2.59	2.35	2.66
40	370	0.21	0.7	0.5	−0.16	−0.2	0.21

The reference ranges: XLogP3: (−2–5), WLogP: (−0.4 to 5.88), and MLogP: (≤ 4.15).

**Table 3:** The LogS values for solubility of propolis compounds predicted using different computation methods’.

S. No.	PubChem ID	Log S (ESOL)	Class	Log *S* (Ali)	Class	Log *S* (SILICOS-IT)	Class
1	44257510	−3.69	Soluble	−3.84	Soluble	−3.69	Moderately soluble
2	21721815	−4.92	Moderately soluble	−5.6	Moderately soluble	−5.55	Moderately soluble
3	11455669	−5.7	Moderately soluble	−6.68	Poorly soluble	−6.78	Poorly soluble
4	11272353	−6.94	Poorly soluble	−8.21	Poorly soluble	−7.75	Poorly soluble
5	11114020	−6.98	Poorly soluble	−8.27	Poorly soluble	−7.75	Poorly soluble
6	10907594	−8.11	Poorly soluble	−10.63	Insoluble	−6.96	Poorly soluble
7	10791588	−7.53	Poorly soluble	−8.8	Poorly soluble	−8.79	Poorly soluble
8	9984117	−6.94	Poorly soluble	−8.21	Poorly soluble	−7.75	Poorly soluble
9	5472440	−4.89	Moderately soluble	−6.33	Poorly soluble	−3.81	Soluble
10	5471610	−8.36	Poorly soluble	−10.52	Insoluble	−8.15	Poorly soluble
11	5377945	−3.36	Soluble	−4.02	Moderately soluble	−3.94	Soluble
12	5352032	−3.96	Soluble	−4.77	Moderately soluble	−4.63	Moderately soluble
13	5318691	−3.66	Soluble	−3.91	Soluble	−5.1	Moderately soluble
14	5281954	−4.39	Moderately soluble	−4.8	Moderately soluble	−5.68	Moderately soluble
15	5281787	−4.24	Moderately soluble	−5.26	Moderately soluble	−4.35	Moderately soluble
16	5281666	−3.51	Soluble	−3.96	Soluble	−4.52	Moderately soluble
17	5281628	−3.99	Soluble	−4.76	Moderately soluble	−4.52	Moderately soluble
18	5281616	−3.46	Soluble	−3.79	Soluble	−4.4	Moderately soluble
19	5280681	−3.89	Soluble	−4.89	Moderately soluble	−3.94	Soluble
20	5280445	−3.71	Soluble	−4.51	Moderately soluble	−3.82	Soluble
21	5280442	−4.14	Moderately soluble	−4.71	Moderately soluble	−5.1	Moderately soluble
22	5280373	−3.92	Soluble	−4.33	Moderately soluble	−5.1	Moderately soluble
23	5280343	−3.16	Soluble	−3.91	Soluble	−3.24	Soluble
24	3938139	−3.81	Soluble	−4.51	Moderately soluble	−3.85	Soluble
25	3873459	−3.48	Soluble	−3.94	Soluble	−4.05	Moderately soluble
26	689043	−1.89	Very soluble	−2.38	Soluble	−0.71	Soluble
27	638278	−3.7	Soluble	−4.48	Moderately soluble	−3.23	Soluble
28	637542	−2.02	Soluble	−2.27	Soluble	−1.28	Soluble
29	637541	−2.22	Soluble	−2.62	Soluble	−1.28	Soluble
30	637540	−2.37	Soluble	−2.87	Soluble	−1.28	Soluble
31	637125	−5.05	Moderately soluble	−6.26	Poorly soluble	−4.02	Moderately soluble
32	637105	−7.77	Poorly soluble	−9.64	Poorly soluble	−8.15	Poorly soluble
33	444539	−2.37	Soluble	−2.54	Soluble	−1.84	Soluble
34	336327	−3.64	Soluble	−3.43	Soluble	−4.31	Moderately soluble
35	259846	−8.64	Poorly soluble	−10.22	Insoluble	−6.74	Poorly soluble
36	238782	−3.64	Soluble	−3.94	Soluble	−4	Soluble
37	92503	−3.69	Soluble	−3.84	Soluble	−4.23	Moderately soluble
38	72307	−3.93	Soluble	−3.5	Soluble	−4.6	Moderately soluble
39	6549	−2.4	Soluble	−3.06	Soluble	−1.84	Soluble
40	370	−1.64	Very soluble	−2.34	Soluble	−0.04	Soluble

ESOL = Estimated Solubility, Log S (Ali) and Log S (SILICOS-IT)= solubility methods developed by Ali and SILICOS-IT, Reference values: insoluble <−10 < poorly <−6 < moderately <−4 < soluble <−2 < very < 0 < highly

**Table 4:** The predicted intestinal absorption, brain permeation and interaction of propolis compounds with cytochromes P450 isoforms’.

S. No.	PubChem ID	GI absorption	BBB permeability	P-gp substrate	CYP1A2 inhibitor	CYP2C19 inhibitor	CYP2C9 inhibitor	CYP2D6 inhibitor	CYP3A4 inhibitor	Log *K*_p_ (skin permeation) cm/s
1	44257510	high	yes	yes	yes	no	No	yes	yes	−5.87
2	21721815	high	yes	no	yes	yes	Yes	yes	yes	−4.94
3	11455669	low	no	yes	no	no	No	no	yes	−6.53
4	11272353	low	no	yes	no	no	No	no	yes	−4.7
5	11114020	low	no	yes	no	no	No	no	yes	−4.66
6	10907594	low	no	yes	no	no	No	no	yes	−3.59
7	10791588	low	no	yes	no	no	No	no	yes	−3.91
8	9984117	low	no	yes	no	no	No	no	yes	−4.7
9	5472440	high	yes	no	no	yes	Yes	no	no	−4.32
10	5471610	low	no	yes	no	no	No	no	yes	−2.89
11	5377945	high	no	no	yes	no	No	yes	yes	−6.9
12	5352032	high	no	no	yes	no	Yes	yes	yes	−6.31
13	5318691	high	no	no	yes	no	No	yes	yes	−6.2
14	5281954	high	yes	no	yes	yes	Yes	yes	yes	−5.2
15	5281787	high	yes	no	yes	no	Yes	no	no	−5.09
16	5281666	high	no	no	yes	no	No	yes	yes	−6.56
17	5281628	high	no	no	yes	no	No	yes	yes	−6.01
18	5281616	high	no	no	yes	no	No	yes	yes	−6.35
19	5280681	high	no	no	yes	no	No	yes	yes	−6.31
20	5280445	high	no	no	yes	no	No	yes	yes	−6.25
21	5280442	high	no	no	yes	no	Yes	yes	yes	−5.66
22	5280373	high	no	no	yes	no	No	yes	yes	−5.91
23	5280343	high	no	no	yes	no	No	yes	yes	−7.05
24	3938139	high	yes	no	yes	no	Yes	no	yes	−5.35
25	3873459	high	yes	no	yes	yes	No	no	no	−5.66
26	689043	high	no	no	no	no	No	no	no	−6.58
27	638278	high	yes	no	yes	no	Yes	no	yes	−5.61
28	637542	high	yes	no	no	no	No	no	no	−6.26
29	637541	high	yes	no	no	no	No	no	no	−6.03
30	637540	high	yes	no	no	no	No	no	no	−5.86
31	637125	high	yes	no	no	yes	Yes	no	yes	−4.09
32	637105	low	no	yes	no	no	No	no	yes	−3.49
33	444539	high	yes	no	no	no	No	no	no	−5.69
34	336327	high	yes	yes	yes	yes	No	yes	yes	−5.98
35	259846	low	no	no	no	no	No	no	no	−1.9
36	238782	high	yes	no	yes	yes	No	no	no	−5.82
37	92503	high	yes	yes	no	no	No	yes	yes	−5.87
38	72307	high	yes	no	no	yes	No	yes	yes	−6.56
39	6549	high	yes	no	no	no	No	no	no	−5.13
40	370	high	no	no	no	no	No	no	yes	−6.84

P-gp = P-glycoprotein, logKp skin permeation, GI = gastrointestinal, BBB = blood brain barrier

**Table 5:** Comparison of drug-likeness of propolis compounds predicted using different methods.

S. No.	PubChem ID	Lipinski rule	Ghose rule	Veber rule	Egan rule	Muegge rule	Bioavailability Score
1	44257510	Yes	Yes	Yes	Yes	Yes	0.55
2	21721815	Yes	Yes	Yes	Yes	Yes	0.55
3	11455669	Yes; 1 violation: MW > 500	Yes; 1 violation: MW > 500	Yes	Yes	Yes	0.56
4	11272353	Yes; 1 violation: MW > 500	No; 4 violations: MW > 480, WLOGP>5.6, MR > 130, #atoms>70	Yes	No; 1 violation: WLOGP>5.8	No; 1 violation: XLOGP3 > 5	0.56
5	11114020	Yes; 1 violation: MW > 500	No; 4 violations: MW > 480, WLOGP>5.6, MR > 130, #atoms>70	Yes	No; 1 violation: WLOGP>5.88	No; 1 violation: XLOGP3 > 5	0.56
6	10907594	Yes; 1 violation: MW > 500	No; 4 violations: MW > 480, WLOGP>5.6, MR > 130, #atoms>70	Yes	No; 1 violation: WLOGP>5.88	No; 1 violation: XLOGP3 > 5	0.56
7	10791588	Yes; 1 violation: MW > 500	No; 4 violations: MW > 480, WLOGP>5.6, MR > 130, #atoms>70	Yes	No; 1 violation: WLOGP>5.88	No; 1 violation: XLOGP3 > 5	0.85
8	9984117	Yes; 1 violation: MW > 500	No; 4 violations: MW > 480, WLOGP>5.6, MR > 130, #atoms>70	Yes	No; 1 violation: WLOGP>5.88	No; 1 violation: XLOGP3 > 5	0.56
9	5472440	Yes; 0 violation	Yes	Yes	Yes	No; 1 violation: XLOGP3 > 5	0.85
10	5471610	Yes; 1 violation: MW > 500	No; 4 violations: MW > 480, WLOGP>5.6, MR > 130, #atoms>70	Yes	No; 1 violation: WLOGP>5.88	No; 1 violation: XLOGP3 > 5	0.85
11	5377945	Yes	Yes	Yes	Yes	Yes	0.55
12	5352032	Yes	Yes	Yes	Yes	Yes	0.55
13	5318691	Yes	Yes	Yes	Yes	Yes	0.55
14	5281954	Yes	Yes	Yes	Yes	Yes	0.55
15	5281787	Yes	Yes	Yes	Yes	Yes	0.55
16	5281666	Yes	Yes	Yes	Yes	Yes	0.55
17	5281628	Yes	Yes	Yes	Yes	Yes	0.55
18	5281616	Yes	Yes	Yes	Yes	Yes	0.55
19	5280681	Yes	Yes	Yes	Yes	Yes	0.55
20	5280445	Yes	Yes	Yes	Yes	Yes	0.55
21	5280442	Yes	Yes	Yes	Yes	Yes	0.55
22	5280373	Yes	Yes	Yes	Yes	Yes	0.55
23	5280343	Yes	Yes	Yes	Yes	Yes	0.55
24	3938139	Yes	Yes	Yes	Yes	Yes	0.55
25	3873459	Yes	Yes	Yes	Yes	Yes	0.55
26	689043	Yes	Yes	Yes	Yes	No; 1 violation: MW < 200	0.56
27	638278	Yes	Yes	Yes	Yes	Yes	0.55
28	637542	Yes	Yes	Yes	Yes	No; 1 violation: MW < 200	0.85
29	637541	Yes	Yes	Yes	Yes	No; 1 violation: MW < 200	0.85
30	637540	Yes	Yes	Yes	Yes	No; 1 violation: MW < 200	0.85
31	637125	No; 1 violation: XLOGP3 > 5	Yes	Yes	Yes	No; 1 violation: XLOGP3 > 5	0.85
32	637105	Yes; 1 violation: MW > 500	No; 4 violations: MW > 480, WLOGP>5.6, MR > 130, #atoms>70	Yes	No; 1 violation: WLOGP>5.88	No; 1 violation: XLOGP3 > 5	0.85
33	444539	Yes	No; 2 violations: MW < 160, #atoms<20	Yes	Yes	No; 1 violation: MW < 200	0.85
34	336327	Yes	Yes	Yes	Yes	Yes	0.55
35	259846	Yes; 1 violation: MLOGP>4.15	No; 3 violations: WLOGP>5.6, MR > 130, #atoms>70	Yes	No; 1 violation: WLOGP>5.88	No; 2 violations: XLOGP3 > 5, Heteroatoms<2	0.55
36	238782	Yes	Yes	Yes	Yes	Yes	0.55
37	92503	Yes	Yes	Yes	Yes	Yes	0.55
38	72307	Yes	Yes	Yes	Yes	Yes	0.55
39	6549	Yes; 0 violation	No; 1 violation: MW < 160	Yes	Yes	No; 2 violations: MW < 200, Heteroatoms<2	0.55
40	370	Yes; 0 violation	No; 2 violations: MR < 40, #atoms<20	Yes	Yes	No; 1 violation: MW < 200	0.56

Bioavailability score (compounds satisfying RO5 with a BS of 0.55 are considered to have excellent oral absorbance).

### iGEMDOCK-based virtual screening of propolis-derived compounds and identification of druggable cavities in the KIFC1 protein: Insights into key molecular interactions via the cavity-binding docking approach

All the propolis compounds examined in this study exhibited significant binding affinities and interactions with the KIFC1 protein (S3 Table). The top ten compounds were selected based on their lowest binding affinity scores. These top ten compounds were then further analyzed from a pool of twenty-two compounds that met all five drug-likeness rules and had a bioavailability score of 0.55. Out of these, the five propolis compounds with the highest binding affinities to KIFC1, namely Luteolin (−92.6849 kcal/mol), Kaempferide (−92.4845 kcal/mol), Izalpinin (−98.6709 kcal/mol), 4’,5,7-Trihydroxy-3,6-dimethoxyflavone (−91.5905 kcal/mol), and 6-Methoxykaempferol (−91.6742 kcal/mol) met all the drug-likeness criteria and had a bioavailability score of 0.55, were chosen for docking studies (**Fig 3**).

The ligand interactions, binding cavities, AutoDock Vina affinity scores, H-bond are summarized in **Table 6**. The molecular docking results showed significant interactions between the KIFC1 protein and the compounds Luteolin, Kaempferide, Izalpinin, 4’,5,7-Trihydroxy-3,6-dimethoxyflavone, and 6-Methoxykaempferol. Notably, Luteolin exhibited the highest binding affinity with cavity C4 (−7.7 kcal/mol) and interacted with ARG470, ALA474, and LYS479, forming H-bonds with GLY480, GLY482, and ASN523. Similarly, Izalpinin showed strong binding affinity with cavity C4 (−7.2 kcal/mol) and interacted with GLU484, CYS485, VAL469, and ARG470, forming H-bond with CYS485 (**Table 6**). Both compounds interacted with ARG470 in cavity C4 showing the highest affinities. However, H-bonds in Luteolin were more prevalent compared to Izalpinin in the respective cavities. However, the docking data of 4’,5,7-Trihydroxy-3,6-dimethoxyflavone, and 6-Methoxykaempferol exhibited their strongest binding toward the ADP‐binding site of KIFC1 and formed H-bond with THR417.

**Table 6:** Analysis of potential druggable cavities in the KIFC1 protein and key molecular interactions with the top-ranked five propolis-derived compounds using the cavity-binding docking approach.

Compound	Cavities ID	Affinity score (kcal/mol)	Cavity volume (Å3)	Center (x, y, z)	Docking size (x, y, z)	Ligand interactions	H-bonds
Luteolin	C4	−7.7	679	2, −2, −31	21, 21, 21	ARG470, ALA474, LYS479, GLY480, GLY482, CYS485, ASN523, ALA527	GLY480, GLY482, ASN523
	C2	−7.4	725	15, −7, −20	21, 21, 21	LEU321, GLY413, SER414, LYS416, THR417, PHE418	LYS416
	C1	−7.3	957	−1, 11, −12	21, 21, 21	GLU496, GLY629, SER631	GLU496, GLY629, SER631
Kaempferide	C1	−7	957	−1, 11, −12	21, 21, 21	GLU496, SER547, CYS557, GLY558, ASN626, LYS673	SER547, ASN626
	C4	−7	679	2, −2, −31	21, 21, 21	ALA474, ARG478, LYS479, CYS485, ASN501	CYS485
	C2	−6.9	725	15, −7, −20	21, 21, 21	ARG318, PRO319, LEU321, GLY413, GLY415, PHE418	GLY413, GLY415
Izalpinin	C4	−7.2	679	2, −2, −31	21, 21, 21	GLU484, CYS485, VAL469, ARG470, ALA474, ALA527	CYS485
	C2	−7.1	725	15, −7, −20	21, 21, 21	ARG318, PRO319, LEU321, GLY413, PHE418, GLY422, PRO424	GLY413
	C1	−6.9	957	−1, 11, −12	21, 21, 21	GLU496, GLN545, PRO560, ASN671	GLN545, ASN671
4’,5,7-Trihydroxy-3,6-dimethoxyflavone	C3	−7	685	26, 3, −29	21, 21, 21	GLU533, ARG537, LEU572, ARG585, THR589, ILE592, ASN593	ARG585, ASN593
	C2	−6.9	725	15, −7, −20	21, 21, 21	LEU321, THR417, PHE418, SER535	THR417
	C4	−6.9	679	2, −2, −31	21, 21, 21	ARG470, ASP471, ALA474, ARG478, LYS479, CYS485, ASN523, VAL526, ALA527	ASP471, ASN523
6-Methoxykaempferol	C2	−7	725	15, −7, −20	21, 21, 21	LEU321, GLY415, THR417, PHE418	THR417
	C1	−6.9	957	−1, 11, −12	21, 21, 21	GLU496, GLN545, PRO560, ASN671	GLN545, ASN671
	C4	−6.9	679	2, −2, −31	21, 21, 21	ARG470, ASP471,ALA474, ARG478, LYS479, GLY480, VAL526, ALA527	ASP471, ARG478, VAL526

### Residue-specific grid-box docking of KIFC1 protein with top-ranked propolis-derived compounds

The 2D protein-ligand plot analysis identified key consensus-binding residues (LEU321, ARG470, ALA474, LYS479, CYS485, and ALA527) present across all five top-ranked propolis-derived compound-based potential druggable cavity identification and their residue-specific interactions with the KIFC protein 3D model. The residue-specific grid-box docking method was employed to analyze the molecular interactions and binding affinities of the key consensus-binding residues within the KIFC1 binding site. A 3D affinity grid box was set with dimensions of 12.34 Å (X), −6.197 Å (Y), and −19.742 Å (Z) to ensure optimal coverage of the identified consensus-binding residues.

**Fig 4:**
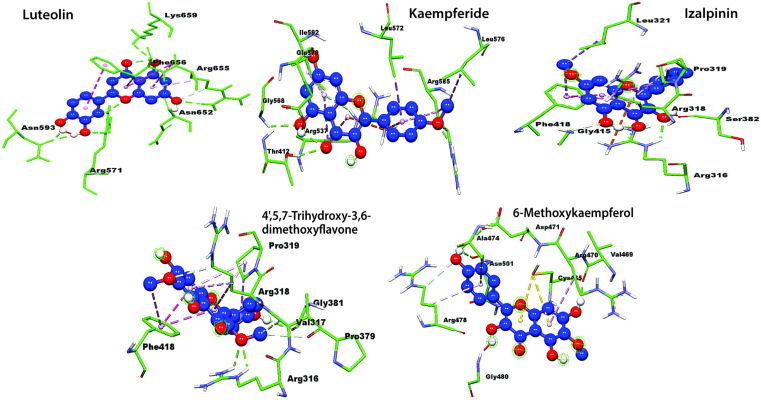
Three dimensional (3D) representations illustrating the binding interactions of the top five propolis-derived compounds with key residues of the KIFC1 protein.

**Fig 5:**
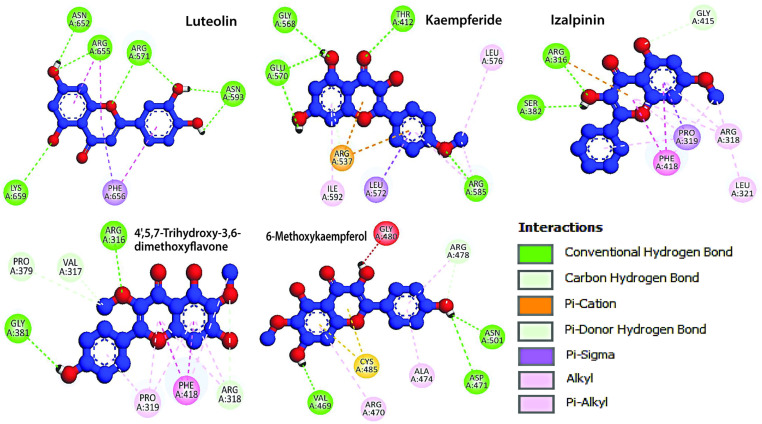
2D interaction diagrams depicting the binding modes of the top five propolis-derived compounds with the KIFC1 protein, highlighting different types of ligands atoms interactions with key protein residues.

**Fig 6:**
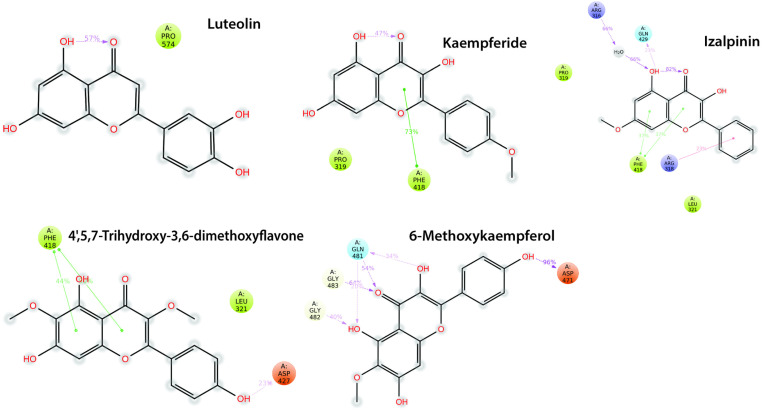
2D interaction diagrams illustrating the binding modes of the top five propolis-derived compounds with the KIFC1 protein, detailing ligand atom interactions with key protein residues. Color scheme: red represents negatively charged residues, light blue indicates polar residues, gray denotes solvent exposure, light green highlights hydrophobic interactions, and dark green signifies Pi-Pi stacking interactions.

All five compounds exhibited ΔG_bind_ between −7.35 and −6.14 kcal/mol (**Table 7**). Figs 4−6 showed the doscking poses (3D and 2D ligand interaction plots) of the inhibitors within the KIFC1 consensus-binding sites, including key residues involved in propolis inhibitor binding. 3D and 2D interaction plots help in the evaluation of binding interaction, depicting the prominent binding modes as well as the different types of molecular bonding and interactions with particular ligands (Fig 4−6). The compound Kaempferide had the lowest *K*_*i*_ value of 4.12 μM, indicating it is the most potent inhibitor among the listed compounds and suggests that it binds most effectively to KIFC1 and inhibits its activity at the lowest concentration followed by Luteolin. Both compounds interacted similarly in terms of electrostatic and torsional energies; however, the stronger van der Waals and molecular mechanics energies of Kaempferide contributed to its significant binding and inhibition efficiency. The compound 4’,5,7-Trihydroxy-3,6-dimethoxyflavone had the highest *K*_*i*_ value of 31.72 μM, indicating it has the less potential of inhibition compared to the rest compounds and requires a higher concentration to achieve the same level of inhibition compared to the other compounds (**Table 7**). Additionally, details of the scientific names along with relevant plant or herbal sources, providing insights into the natural origins and potential therapeutic relevance of the five selected propolis-derived compounds based on their docking scores for their selection, are presented in **Table 8**.

**Table 7:** Residue-specific grid-box docking of KIFC1 protein with top-ranked propolis-derived compounds and estimation of inhibition constant for protein-ligand complexes.

Compounds	ΔG_bind_ (kcal/mol)	Ligand efficiency	Inhibition constant (μM)	ΔE_vdw_	ΔE_ele_	ΔE_MM_	ΔE_(unbound)_	ΔE_(torsional)_	ΔE_(total internal)_	ΔE_(intermolecular)_
Luteolin	−6.74	−0.32	11.48	−7.67	−0.44	−8.11	−1.77	1.37	−1.77	−8.11
Kaempferide	−7.35	−0.33	4.12	−8.27	−0.44	−8.71	−1.32	1.37	−1.32	−8.72
Izlapinin	−6.33	−0.3	22.9	−7.24	−0.18	−7.46	−1.05	1.1	−1.05	−7.43
4’,5,7-Trihydroxy-3,6-dimethoxyflavone	−6.14	−0.26	31.72	−7.56	−0.23	−7.79	−1.72	1.65	−1.72	−7.78
6-Methoxykaempferol	−6.55	−0.28	15.81	−8	−0.2	−8.2	−1.71	1.65	−1.71	−8.2

ΔG_bind_ = Binding Affinity, ΔE_vdw_ = van der Waals energy, ΔE_ele _= electrostatic energy, = ΔE_MM *=*_molecular mechanics energy

**Table 8:** Top-ranked propolis-derived compounds from molecular docking: Scientific names, natural sources, and therapeutic significance.

Compound Name	Scientific Name	Compound Natural and Source	Potential Therapeutic Relevance	References
Kaempferol	3,5,7-trihydroxy-2-(4-hydroxyphenyl)-4H-1-benzopyran-4-one	Flavonoid – found in propolis, tea, broccoli, apples, kale, beans, tomato, strawberries, grapes and medicinal herbs	Cardio-protective, anticancer, antidiabetics, anti-inflammatory, Osteo-protective.	[71–73]
Luteolin	3′,4′,5,7-tetrahydroxyflavone	Flavonoid -Present in propolis, celery, spinach, lettuce, sweet and chili peppers	Known for its anticancer, anti-inflammatory, anti-oxidant, and neuroprotective effects. It interferes with key signaling pathways involved in tumor growth and metastasis.	[74,75]
Kaempferide	Kaempferol 4’-O-methyl ether	Flavonoid - Found in propolis and various medicinal plants such as Kaempferia species	Demonstrates anticancer activity by targeting multiple signaling pathways involved in cancer progression. Anti-inflammatory, anti-adipogenic, antioxidant, immune modulation, etc	[76]
Izalpinin	3,5-Dihydroxy-7-methoxyflavone	Flavonoid - Found in propolis and medicinal plants such as Alpinia species	Exhibits antimicrobial, anti-inflammatory, and anticancer properties.	[77,78]
4’,5,7-Trihydroxy-3, 6-imethoxyflavone	6-Methoxykaempferol 3-methyl ether	Flavonoid - Isolated from propolis and selected medicinal herbs	Potential anticancer agent with reported inhibition of cancer cell migration and proliferation. Also known for its antioxidant, antiepileptic and anticholinergic effects.	[11,79]

## Molecular dynamics simulation

Molecular dynamics (MD) simulations were conducted for 100 ns to evaluate the stability and binding interactions of KIFC1 in complex with Luteolin, Kaempferide, Izalpinin, 4’,5,7-Trihydroxy-3,6-dimethoxyflavone, and 6-Methoxykaempferol. Each system consisted of approximately 58,260 atoms, including a well-hydrated environment, to ensure the system stability. The respective numbers of water molecules were 17,389 for Luteolin, 17,388 for Kaempferide, 17,386 for Izalpinin, 17,384 for 4’,5,7-Trihydroxy-3,6-dimethoxyflavone, and 17,386 for 6-Methoxykaempferol. The solvation process ensured a biologically relevant environment throughout the simulations.

**Fig 7:**
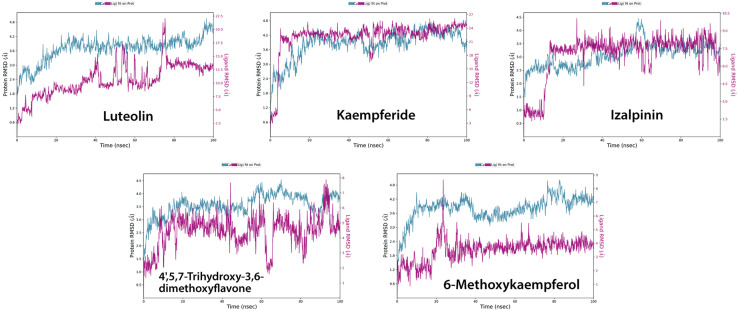
Time dependent variation in the root mean square deviation (RMSD) between the C-alpha atoms (in blue) of proteins and ligands fit on proteins (red) over time of top-ranked propolis compounds with KIFC1 protein. The protein RMSD shifts over time are plotted on the left Y axis. Differences in the ligand root-mean-square distance (RMSD) over time are plotted along the right Y-axis.

The average atom displacement between two superimposed molecular structures is measured quantitatively by RMSD (root mean square deviation), which is frequently used in molecular docking and structural biology to evaluate conformational differences. **Fig 7** shows the time-dependent changes in the root-mean-square deviation (RMSD) values for the C-alpha atoms of ligand-bound KIFC1, confirming the structural stability of the complexes. The fluctuations remained within an acceptable range of ~1.2 to 3.5 Å. In the Luteolin-bound system, the protein remained stable throughout the simulation, except for a minor fluctuation observed at 75 ns, which quickly stabilized at approximately 80 ns. The Kaempferide-KIFC1 complex exhibited RMSD values within ~1.8 to 4.2 Å, with a stable pattern emerging at 10 ns and persisting throughout the simulation. The Izalpinin-protein complex remained stable within a ~ 2–3.5 Å range with some minor fluctuations at 60 ns, which became stable immediately, with the ligand RMSD remaining relatively stable across the trajectory, indicating an equilibrated system. The 4’,5,7-Trihydroxy-3,6-dimethoxyflavone-KIFC1 complex maintained a stable RMSD range of 2 to 3.5 Å, demonstrating that the ligand remained firmly bound to the druggable binding site. The 6-Methoxykaempferol complex exhibited fluctuations within ~1.8 to 3.6 Å, with a minor variation observed after 20 ns that quickly stabilized. This consistency confirms the stability of the ligand within the druggable binding site (**Fig 7**).

**Fig 8:**
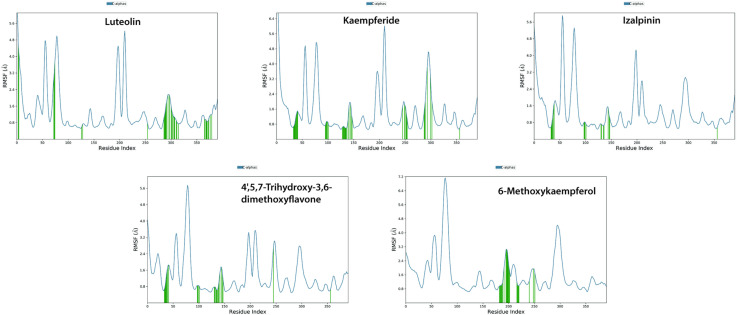
The root mean square fluctuation (RMSF) plot of protein (KIFC1) and ligands (top-ranked propolis compounds) based on C α atoms of receptor proteins. Protein residues that interacted with the propolis compounds are marked with green vertical bars.

The RMSF values of the ligands were further analyzed to evaluate their stability in druggable binding modes of the protein (**Fig 8**). The peaks indicate the protein regions that fluctuated the most during the simulation, and the green vertical bars show ligand contacts with KIFC1 protein residues that interact with the ligands. Analysis of the RMSF MD trajectories showed high fluctuations in the loop region or the N- and C-terminal zones of the protein (**Fig 8**). The low RMSF values of the binding site residues indicate a stable ligand-protein complex. The RMSF plots of 4’,5,7-Trihydroxy-3,6-dimethoxyflavone and 6-methoxykaempferol revealed structural stability variations with varying flexibilities at different residue indices. Luteolin, Kaempferide and Izalpinin showed significant deviations at specific residues, affecting overall stability of ligands in binding mode (**Fig 8**).

**Fig 9:**
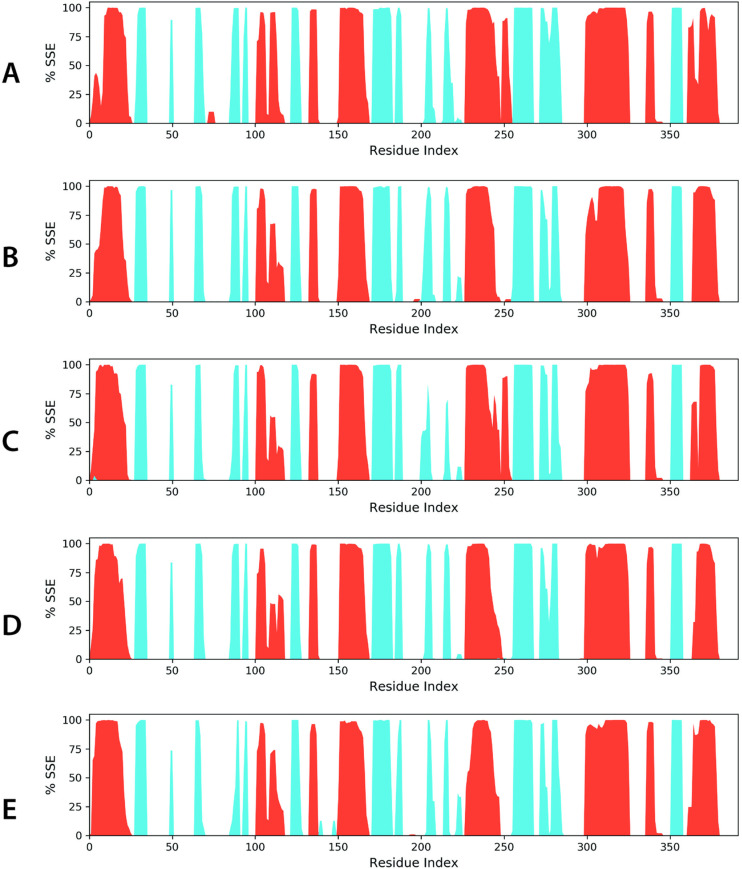
The distribution of secondary structure elements (SSE) across the protein-ligand complexes is illustrated for **(A)** **Luteolin,** **(B)** **Kaempferide,** **(C)** **Izalpinin, (D) 4’,5,7-Trihydroxy-3,6-dimethoxyflavone, and (E) 6-Methoxykaempferol, mapped against the residue index.** Alpha-helices are depicted as red columns, while beta-strands are shown in blue, highlighting the structural organization within each complex.

To assess the structural stability, the secondary structure elements (SSE) were analyzed over the simulation period (**Fig 9**). The SSE content remained consistent, with minor deviations: luteolin, 50.16% (29.61% helices, 20.56% strands); Kaempferide, 47.20% (27.18% helices, 20.02% strands); Izalpinin, 47.88% (28.84% helices, 19.04% strands); 4’,5,7-Trihydroxy-3,6-dimethoxyflavone: 47.64% (27.76% helices, 19.88% strands), 6-Methoxykaempferol: 47.16% (28.31% helices, 18.85% strands) (**Fig 9**). These findings indicated that ligand binding does not induce significant structural distortions in KIFC1.

**Fig 10:**
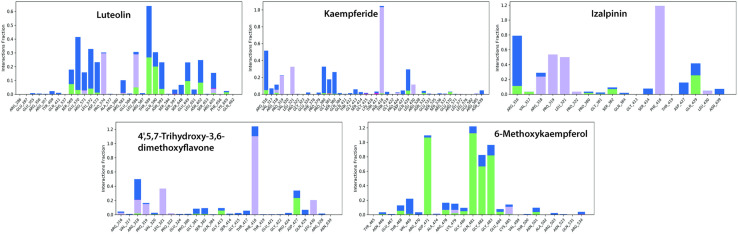
Heat-map representation of protein-ligand interactions throughout the simulation trajectory for (A) Luteolin, (B) Kaempferide, (C) Izalpinin, (D) 4’,5,7-Trihydroxy-3,6-dimethoxyflavone, and (E) 6-Methoxykaempferol, mapped against the residue index.

The key molecular interactions identified through MD simulations are illustrated in **Fig 10**, detailing the hydrogen bonds, hydrophobic contacts, and water-mediated interactions. Luteolin formed stable hydrogen bonds with SER569, GLU570, LEU572, LEU586, THR589, GLN590, GLU649, and ASN652. Hydrophobic interactions with ARG571, PRO574, LEU586, and ARG655 further reinforce ligand stability. Water-mediated hydrogen bonding significantly contributed to ligand retention. Kaempferide forms hydrogen bonds with ARG316, ARG318, PRO380, SER382, PHE418, GLN429, ASN532, and GLU570. Hydrophobic contacts with ARG318, PRO319, LEU321, PHE418, and LEU430 stabilized the ligand within the pocket, while water bridges enhanced binding stability. Izalpinin-KIFC1 interactions established hydrogen bonds with ARG316, VAL317, PRO380, GLY381, SER382, and GLN429, which contributed to ligand stability. Hydrophobic interactions with ARG318, PRO319, LEU321, PRO322, PHE418, and LEU430 provided additional support, whereas water bridges reinforced the retention of the ligand in the active site. 4’,5,7-Trihydroxy-3,6-dimethoxyflavone-KIFC1 interactions formed stable hydrogen bonds with ARG318, GLY381, SER382, GLY413, PRO424, and ASP427. Hydrophobic interactions with ARG316, ARG318, PRO319, LEU321, PRO322, PHE418, and LEU430 stabilized the ligand with minimal ionic interactions observed. Water-mediated hydrogen bonding further reinforced this complex. 6-Methoxykaempferol-KIFC1 interactions exhibited hydrogen bonds with ASN466, THR468, VAL469, ASP471, ARG478, LYS479, GLY480, GLN481, GLY482, GLY483, GLU484, and ASN501, ensuring stable ligand retention. Hydrophobic interactions with LYS479 and CYS485 contributed to ligand stability, whereas water-mediated hydrogen bonds maintained strong binding (**Fig 10**).

Overall, MD simulation experiments confirmed that all five ligands established stable interactions with KIFC1, demonstrating strong binding affinity without inducing significant structural distortions. These findings provide valuable insights into the molecular mechanisms governing ligand binding, and highlight the potential of these compounds for further experimental validation.

## Principal component analysis

**Fig 11:**
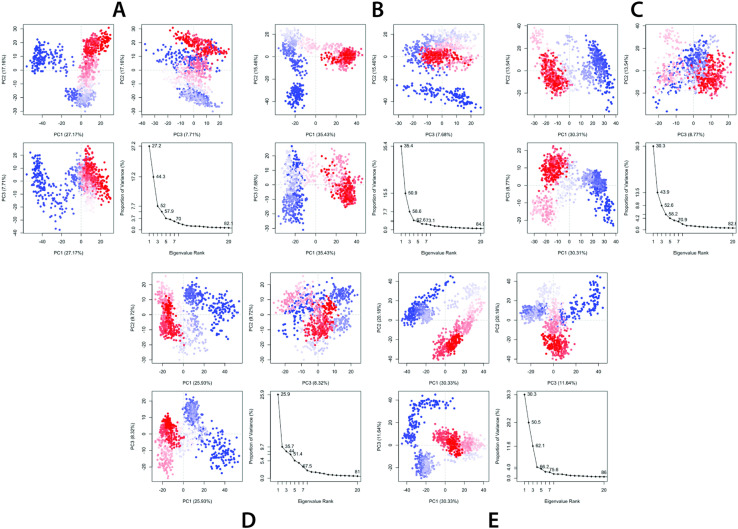
Principal Component Analysis (PCA) plot illustrating the percentage of variance across different principal components (PC1, PC2, and PC3). Three distinct sections were generated to observe variations in molecular dynamics. The analysis was performed for (A) Luteolin, (B) Kaempferide, (C) Izalpinin, (D) 4’,5,7-Trihydroxy-3,6-dimethoxyflavone, and (E) 6-Methoxykaempferol.

Principal Component Analysis (PCA) is an essential technique used in molecular dynamics (MD) simulations to analyze the collective motions of proteins. It reduces the complex motion of atoms into selective principal components (PCs) to recognize the dominant conformational changes occurring during the simulation. In this study, PCA was performed on KIFC1-ligand complexes to evaluate the structural stability, flexibility, and dynamic behavior of the system when bound to luteolin (A), kaempferide (B), Izalpinin (C), 4’,5,7-Trihydroxy-3,6-dimethoxyflavone (D), and 6-Methoxykaempferol (E) (**Fig 11**). The collective motions of the protein were analyzed throughout the MD simulation to capture dynamic fluctuations over the trajectory. The eigenvalue plots against the eigenvector index (eigenmode) confirm the stability of the system (**Fig 11**). The PCA trajectories of ligands A, B, C, D, and E demonstrated distinct influences on the conformational landscape of KIFC1, highlighting the variations in structural flexibility. Fluctuations in hyperspace eigenvectors were observed within the eigenvalues, where higher eigenvalues were correlated with increased protein mobility. Eigenvector analysis indicated that dominant motions were present across all the selected complexes, with higher eigenvalues reflecting significant conformational shifts. To interpret these variations further, three principal components (PC1, PC2, and PC3) were extracted and plotted. PC3 exhibited the least variability (**Fig 11**), suggesting a more compact and stabilized protein-ligand complex. PC1 clusters of Kaempferide (B) compound had exhibited the highest variability (35.43%), PC2 showed variability (15.46%), and PC3 had the lowest variability (7.68%). Overall, the reduced variability in PC3 cells supports the understanding that ligand binding reinforces structural integrity, limiting excessive motion. Additionally, the color-coded PCA plot provided a visual representation of protein flexibility: blue areas corresponded to high mobility, white areas indicated intermediate motion, and red areas represented minimal flexibility (**Fig 11**).

### MM-GBSA binding free energy analysis of KIFC1 with propolis-derived compounds

MM-GBSA is a widely used computational approach to estimate the binding free energies (ΔG Bind) of protein-ligand complexes. In this case, computation with lower ΔG Bind values indicated stronger binding affinities between the ligand and the target protein. The MM-GBSA binding free energies (ΔG Bind) of the KIFC1-ligand complexes showed significant pattern of energetic estimation like KIFC1-Luteolin, i.e.*,* −39.8675 kcal/mol, KIFC1-Kaempferide, i.e.*,* −33.8669 kcal/mol, KIFC1-Izalpinin, i.e.*,* −44.4599 kcal/mol, KIFC1–4’,5,7-Trihydroxy-3,6-dimethoxyflavone, i.e.*,* −42.003 kcal/mol and KIFC1–6-Methoxykaempferol, i.e.*,* −53.0327 kcal/mol (**Table 9**). Among these five compounds, 6-Methoxykaempferol exhibited the strongest binding affinity (−53.0327 kcal/mol), followed by Izalpinin (−44.4599 kcal/mol), and 4’,5,7-Trihydroxy-3,6-dimethoxyflavone (−42.003 kcal/mol). Luteolin (−39.8675 kcal/mol) and Kaempferide (−33.8669 kcal/mol) also showed favorable interactions but with slightly weaker binding affinities compared to the other top-ranked propolis-derived compounds (**Table 9**).

**Table 9:** MM-GBSA binding free energies computations of the top-ranked docked KIFC1-ligand (propolis-derived small molecules) complexes.

Energies	KIFC1-Luteolin	KIFC1- Kaempferide	KIFC1- Izalpinin	KIFC1–4’,5,7-Trihydroxy-3,6-dimethoxyflavone	KIFC1–6-Methoxykaempferol
**MM-GBSA** **ΔG Bind (kcal/mol)**	−39.8675	−33.8669	−44.4599	−42.003	−53.0327
**MM-GBSA ∆G** **Bind Coulomb (kcal/mol)**	−12.4349	−14.6728	−0.77943	−17.9378	−28.2251
**MM-GBSA ∆G** **Bind Covalent** **(kcal/mol)**	1.583922	0.439944	−0.22495	1.896344	0.84432
**MM-GBSA ∆G** **Bind H-bond** **(kcal/mol)**	−0.91523	−0.0000921	−0.76707	−0.38889	−3.48526
**MM-GBSA** **∆G Bind Lipo** **(kcal/mol)**	−5.07359	−6.16688	−10.0106	−12.0955	−7.51317
**MM-GBSA** **∆G Bind Solv** **GB (kcal/mol)**	10.44199	19.53683	9.145694	21.87094	21.01219
**MM-GBSA** **∆G Bind** **vdW (kcal/mol)**	−27.8384	−27.5436	−36.2582	−31.2444	−33.2059

The description of above energies are indicated as Coulomb—Coulomb energy; Covalent—Covalent binding energy; H-bond—Hydrogen-bonding; Lipo—Lipophilic energy; Solv GB—Generalized Born electrostatic solvation energy and vdW—Van der Waals energy.

The MM-GBSA binding free energy is influenced by several contributing factors, including Coulombic interactions, covalent binding energy, hydrogen bonding, lipophilic interactions, solvation energy, and van der Waals (vdW) forces, which were also computed. The strongest electrostatic contribution was observed for 6-Methoxykaempferol (−28.2251 kcal/mol), followed by 4’,5,7-Trihydroxy-3,6-dimethoxyflavone (−17.9378 kcal/mol). In contrast, Izalpinin (−0.77943 kcal/mol) had significantly lower Coulomb interactions, suggesting a lower contribution from electrostatic forces. The highest hydrogen bonding energy was recorded for 6-Methoxykaempferol (−3.48526 kcal/mol), followed by luteolin (−0.91523 kcal/mol), and Izalpinin (−0.76707 kcal/mol). Izalpinin (−10.0106 kcal/mol) and 4’,5,7-Trihydroxy-3,6-dimethoxyflavone (−12.0955 kcal/mol) showed strong lipophilic interactions, which can enhance ligand retention in the binding pocket. Kaempferide (−6.16688 kcal/mol) had the weakest lipophilic contribution among the five ligands. Izalpinin (−36.2582 kcal/mol) exhibited the strongest vdW interactions, followed by 6-Methoxykaempferol (−33.2059 kcal/mol) (**Table 9**). These interactions play a crucial role in maintaining ligand stability within the KIFC1 binding pocket.

## Discussion

Kinesin family member C1 (KIFC1) is crucial for cell division and highly expressed in cancer cells. KIFC1 plays an essential role in centrosome clustering, a process that allows cancer cells to bypass multipolar divisions and maintain genomic stability. By crosslinking microtubules and facilitating bipolar spindle formation, KIFC1 prevents chromosomal missegregation, which is a key factor in tumor survival. Additionally, its interactions with mitotic regulators, such as cyclins and spindle checkpoint proteins, further underline its importance in sustaining the proliferative potential of malignant cells. Given that normal somatic cells do not rely on KIFC1 for spindle organization, its targeted inhibition presents a promising strategy for selectively disrupting cancer cell [18,25,30–35,80]. However, drugs targeting KIFC1 inhibition remain limited. Propolis is known to inhibit cancer cell proliferation, angiogenesis, metastasis and as adjunct chemotherapeutic agent [25,80,81]. This study was designed to explore propolis-derived small molecules as KIFC1 inhibitors for cancer therapy using an *in silico* approach.

Protein homology modeling predicts a protein’s three-dimensional structure using amino acid sequences and reduces the time, labor, and cost associated with traditional experimental techniques used for drug design[80]. The results of KIFC1 protein sequence alignment methods provided models with less than 60% coverage in the Protein Data Bank (PDB). Therefore, AlphaFold 3D model was preferred to achieve accurate protein structure prediction because the AlphaFold-generated models undergo rigorous energy minimization, structural assessment, and validation processes, ensuring their precision and reliability [42,44]. In the Critical Assessment of Techniques for Protein Structure Prediction (CASP14), AlphaFold was the top-ranked protein structure prediction method by a large margin, producing predictions with high accuracy established by Google DeepMind and EMBL’s European Bioinformatics Institute (EMBL-EBI) [42]. Furthermore, the stereo-chemical assessment and validation for selected AlphaFold 3D model further provided a justification of KIFC1 for subsequent experiment [48]. It is established that computer aided pharmacokinetics predictions are cost-effective and save resources in the new drug design and development in contrast to experimental studies. The study used Lipinski’s rule of five which is critical for rational drug design and low permeability or poor absorption of a specific molecule occurs when it violates one of Lipinski’s rule of fives [82,83]. The molecular weights of the most propolis-derived compounds were less than 500 and TPSA was less than 150 predicting adequate absorption. The lipohilicity and water solubility parameters of these compounds showed a balance of lipohilicity and hydrophilicity indicating favorable ADME properties. These findings are in agreement with the studies that predicted the ADMET properties and conducted *in vivo*/ *in vitro* experiments for further validation of outcomes [82]. The CYP3A4 inhibitors have the potential to enhance the effectiveness of specific chemotherapy drugs by increasing their plasma concentrations and bioavailability [52,82]. The top ten conformations for each compound (ligand) based on binding affinity energy were saved and the top‐5 binding poses were selected from an initial set of forty compounds. Based on drug-likeness criteria and binding affinity with the KIFC1 protein, 6-methoxy kaempferol, 4’,5,7-Trihydroxy-3,6-dimethoxyflavone, Izalpinin, Kaempferide, and luteolin were identified as the best candidates. All the five compounds showed a similar bioavailability score (0.55) and inhibited the enzymes CYPIA2, CYP2D6, and CYP3A4, with 4’,5,7-Trihydroxy-3,6-dimethoxyflavone also inhibiting CYP2C9, indicating their potential as drug candidates for KIFC1 inhibition. Kaempferol, Luteolin, Kaempferide, Izlapinin and 4’,5,7-Trihydroxy-3, 6-dimethoxyflavone are flavonoid known to inhibit tumor growth by targeting various cellular processes such as apoptosis, angiogenesis, migration, and cell cycle progression [42,62,73,84,85].

Residue-specific grid-box based cavity binding (docking) allowed precise identification and evaluation of the interaction sites between the five propolis-derived compounds and KIFC1. This method helps predict the binding affinity and stability of drug candidates [58,86]. The molecular docking results revealed significant interactions between the KIFC1 protein and the five selected compounds. Notably, Luteolin demonstrated the highest binding affinity to cavity C4, closely followed by Izalpinin. Both Luteolin and Izalpinin displayed the highest affinity for ARG470 in cavity C4. Luteolin, in particular, formed more hydrogen bonds compared to Izalpinin in the respective cavities. These interactions with ARG470 in both compounds highlight its critical role in binding efficacy, indicating its potential as a key target for designing KIFC1 inhibitors. These results reinforce the significance of hydrogen bonding and specific residue interactions in enhancing binding affinity and stability, in agreement with previous studies [87–89]. However, the grid-box molecular interactions and binding analysis predicted Kaempferide, with a *K*_*i*_ of 4.12 μM, and binding energy of −7.35 kcal/mol as the most significant inhibitor which was comparable to the most effective KIFC1 inhibitor AZ82 (binding energy of −7.26 kcal/mol) [36].

Molecular dynamics (MD) simulations are important for designing novel drugs because they provide insights into the behavior and interactions of drug molecules with target proteins or biological systems [90]. We employed this method to analyze the five commonly interacting top-ranked docked complexes to understand the function of small molecules derived from propolis in inhibiting the KIFC1 protein, thus controlling the KIFC1 pathway to regulate cancer expression. The general stability of the protein-ligand complexes was evaluated by calculating the root‐mean‐square deviation (RMSD). The RMSDs for both 4’,5,7-trihydroxy-3,6-dimethoxyflavone and 6-methoxy kaempferol complex with KIFC converged during the 100 ns in a range of 1.5–3.9 Å. These findings represent quality predictions based on a critical assessment of the prediction of interaction criteria, and demonstrate high stability. The observed minor time interval-associated variations in RMSD values are common in molecular dynamics simulations and may not necessarily impact the overall stability or efficacy of the complexes [91,92].

RMSF analysis provides dynamic perspective on protein flexibility and focuses on the regions that are crucial for conformational changes and binding events, whereas the distribution of secondary structure elements (SSEs) helps to understand how ligand binding affects the overall structure and stability of proteins [93]. The RMSF analysis of two selected protein-ligand complexes in this study showed that upon binding to the 4’,5,7-trihydroxy-3,6-dimethoxyflavone and 6-methoxy kaempferol, the highest molecular motion was observed in residues that were not present in the active site or substrate-binding region of KIFC1 protein. These RMSF results indicate that the propolis compounds are stabilized in the protein-binding regions. SSE analysis revealed that the structural integrity of KIFC1 was preserved by both ligands, although minute differences in the strand and helix composition may have an effect on biological activity. It was specifically observed that after simulation, PHE418 formed hydrophobic and water bridges with 4’,5,7-trihydroxy-3,6-dimethoxyflavone in addition to its binding to the ADP site of KIFC1. This binding combined with the highest docking score improves the potency and physicochemical properties of the compound and fits it well into the binding pocket through Pi-Pi stacking interactions with PHE418. Therefore, this study suggests that 4’,5,7-trihydroxy-3,6-dimethoxyflavone, a natural compound with drug-likeness and favorable physicochemical properties, is a promising lead for further optimization; however, further cell assays and clinical validation studies are needed.

MM-GBSA analysis provided a detailed understanding of the binding energetics of the five propolis-derived ligands with KIFC1 [70]. 6-Methoxykaempferol has emerged as the most promising inhibitor, exhibiting the strongest binding affinity (−53.0327 kcal/mol) due to its high electrostatic, hydrogen bonding, and van der Waals contributions. Izalpinin (−44.4599 kcal/mol) and 4’,5,7-Trihydroxy-3,6-dimethoxyflavone (−42.003 kcal/mol) also demonstrated favorable interactions, making them strong candidates for further experimental validation. Luteolin (−39.8675 kcal/mol) and Kaempferide (−33.8669 kcal/mol) showed moderate binding affinities, suggesting potential for structural optimization to improve their interactions with KIFC1. These findings emphasize the potential of 6-Methoxykaempferol, Izalpinin, and 4’,5,7-Trihydroxy-3,6-dimethoxyflavone as possible lead compounds for the synthesis of similar drug-like bio-active compounds having optimal physicochemical properties and bioavailability for targeting KIFC1 inhibition, paving the way for further refinement and *in vitro* validation of anticancer drug development.

## Conclusion

KIFC1 is crucial for centrosome clustering, prevention of chromosomal missegregation, and maintenance of genomic stability. It interacts with mitotic regulators to sustain the proliferative potential of malignant cells. Targeting KIFC1 inhibition could disrupt cancer cell division, and propolis-derived small molecules are being explored as potential inhibitors. The present computational analysis of selected propolis-derived compounds as potential KIFC1 inhibitors revealed that 4’,5,7-trihydroxy-3,6-dimethoxyflavone and 6-methoxy kaempferol may be promising candidates for cancer therapy. This study attempted to provide a thorough analysis of propolis-derived compounds using computational benchmarking for KIFC1 inhibition. This may help overcome the challenges of transitioning these compounds from bench to bedside, reduce toxicity, and improve patient outcomes in cancer therapy after clinical validation.

## Supporting information

S1 Table Sequence alignment and protein-protein Basic Local Alignment (pBLAST) search of selected KIFC1 proteins of *Homo sapiens.* (XLSX)

S2 Table List of the compounds selected from propolis. (XLSX)

S3 Table Virtual screening through graphical-automatic drug design (iGEMDOCK) approach and post-screening analysis of propolis compounds’ complexes with KIFC1. (XLSX)

S1 Fig Ramachandran plot of KIFC1 protein retrieved from AlphaFold database. (DOCX)
