# Supplementary figures and images for: KIFC1 inhibition: Exploring the potential of propolis-derived small molecules for targeting cancer progression through in silico analysis
Source: PLoS One. 2025 Jun 5;20(6):e0324678. doi: 10.1371/journal.pone.0324678 (PMC12140393; doi:10.1371/journal.pone.0324678)

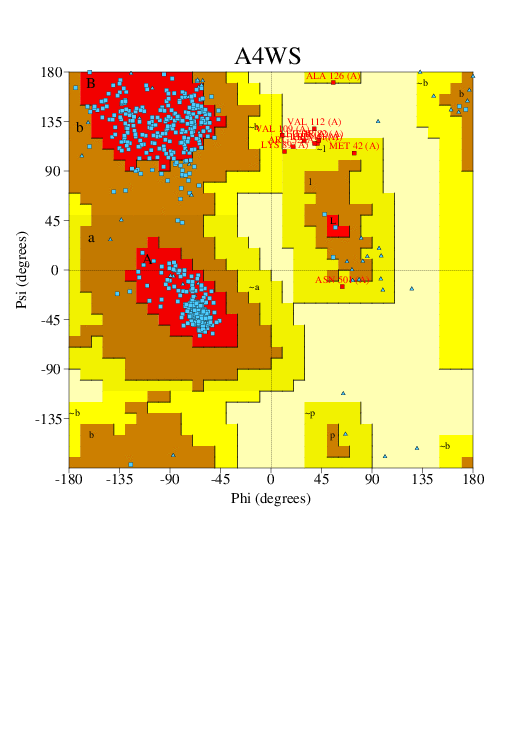


**S1 Fig. Ramachandran plot of KIFC1 protein retrieved from AlphaFold database.**

Supplement: S1 Fig — (DOCX) [file pone.0324678.s004.docx]
